# Gene Therapy - Can it Cure Type 1 Diabetes?

**DOI:** 10.7759/cureus.20516

**Published:** 2021-12-19

**Authors:** Mirra Srinivasan, Santhosh Raja Thangaraj, Hadia Arzoun

**Affiliations:** 1 Internal Medicine, California Institute of Behavioral Neurosciences & Psychology, Fairfield, USA

**Keywords:** complications, autoimmune disease, pancreatic β-cells, insulin gene therapy, type 1 diabetes, gene therapy

## Abstract

Type 1 diabetes (T1D) is one of the most prevalent early-onset autoimmune diseases, and numerous treatment regimens have been developed over the years with a mainstay focus on insulin injections, infusions, and pumps. However, with the evolution of modern medicine in the recent decade, can gene therapy be a possible solution to prevent and even cure this autoimmune diabetes? In this review, the authors discuss the present-day advancements around the globe where gene therapy is implemented in different techniques to halt and even reverse T1D. The main focus of the final included studies for this review was to regenerate or preserve pancreatic β cells from other cell types in order to optimize insulin secretions in non-obese autoimmune diabetic patients. A literature search was done in various databases such as PubMed, ScienceDirect, and Google Scholar, and a final of eight studies were included. On the whole, the studies reviewed suggested favorable results of gene therapy, although these researches were done mainly in vitro or as animal studies. The application of different virus vector encoding gene transfer through transcription factors, mRNA electroporation, insulin-like growth factor gene expression as well as combination gene transfer concluded beneficial effects on normalizing insulin production, which could pave the path to perfecting gene therapy, and may even find a permanent cure for T1D in the near future.

## Introduction and background

Type 1 diabetes (T1D) is a T-cell mediated autoimmune disease where one's pancreatic β-cells fail to produce insulin [1], without which the consumed glucose, which is the primary source of energy of all living cells, does not enter the cells, instead starts to accumulate in the blood vessels, subsequently leading to high blood glucose levels [2]. The elevated blood glucose, beyond the threshold, manifests as varying symptoms that begin in childhood and ultimately lead to multisystem complications such as hyperglycemia, diabetic ketoacidosis (DKA), and psychiatric disorders, as well as long-term sequelae such as retinopathy, nephropathy, neuropathy, and cardiovascular disease [3]. However, clinical manifestations of these complications are uncommon before adulthood [2]. At present, no definitive prevention or cure exists for T1D, and most treatments only try to treat the symptoms and prevent complications using various regimens of insulin injections/pumps [1] and dual-hormone artificial pancreas systems that deliver supplemental hormones (glucagon or amylin) in addition to insulin. Studies have shown to mimic the physiology of the endocrine pancreas better and have been proposed as an alternative approach in managing T1D [4]. Recent research has started looking into gene therapy as an answer to restore the deranged insulin-glucose metabolism in patients with T1D [5].

Gene therapy is an approach to treat diseases by remodeling one's deoxyribonucleic acid (DNA) that functions in a variety of ways: 1) replacing a disease-causing gene with a healthy copy of the gene, 2) inactivating a disease-causing gene that isn't functioning as expected, or 3) introducing a new or modified gene into the body to aid in disease treatment. Cancer, hereditary/autoimmune illnesses, and infectious diseases are common disorders where gene therapy is currently being researched. Plasmid DNA, viral vectors, bacterial vectors, human gene-editing technology, and patient-derived cellular gene therapy products are the different types of gene therapy [6].

The University of Wisconsin School of Medicine and Public Health is the first to validate a DNA-based insulin gene therapy that could possibly treat T1D. They injected a small sequence of DNA into the veins of diabetic rats, which then created insulin-producing cells that regulated blood sugar levels, in turn normalizing the glucose metabolism [6], thus opening doors for further research in this domain.

The study group in the selected reports included animals such as mice and bone marrow aspirate from healthy canines. Gene therapy was used in the intervention group in the trials included for this study, and it was administered using various methods, as detailed below. The purpose of this review is to comprehend the recent trends of gene therapy in T1D while also looking beyond the scope to make this novel therapy a possibility to patients around the globe, ultimately achieving a global cure.

Methods

The authors explored the following databases, namely PubMed, Science Direct, and Google Scholar, to address the research question at hand. After employing the search strategy with the regular and Medical Subject Headings (MeSH) keywords using the Boolean scheme mentioned below, yielded 792 results, and articles published after 2015 in the English language were included. The final eight studies selected for this review consisted of original animal and human (preclinical) studies. The data selection and extraction were carried out independently by two researchers. When the authors couldn't agree, they discussed the study designs, inclusion and exclusion criteria, intervention employed, and results measured. In case of discrepancies between the authors, a third reviewer was approached to help resolve disagreements and find common ground.

Keywords

MeSH Keywords

Gene therapy OR insulin gene therapy OR ("genetic therapy/methods"[MeSH] OR "genetic therapy/therapeutic use"[MeSH]) AND type 1 diabetes OR pancreatic β-cells OR ("diabetes mellitus, type 1/complications"[MeSH] OR "diabetes mellitus, type 1/drug therapy"[MeSH] OR "diabetes mellitus, type 1/genetics"[MeSH] OR "diabetes mellitus, type 1/metabolism"[MeSH] OR "diabetes mellitus, type 1/prevention and control"[MeSH])

Keywords on Other Databases

Gene therapy; type 1 diabetes; insulin gene therapy; pancreatic β-cells; autoimmune disease; complications

**Figure 1:**
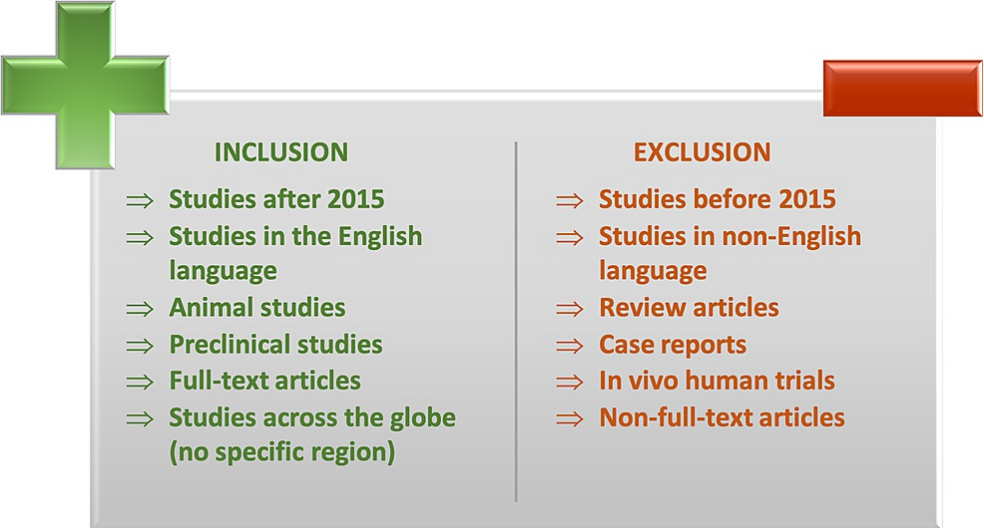
Inclusion and exclusion criteria

Figure 1 depicts the inclusion and exclusion criteria for this review.

Results

**Figure 2:**
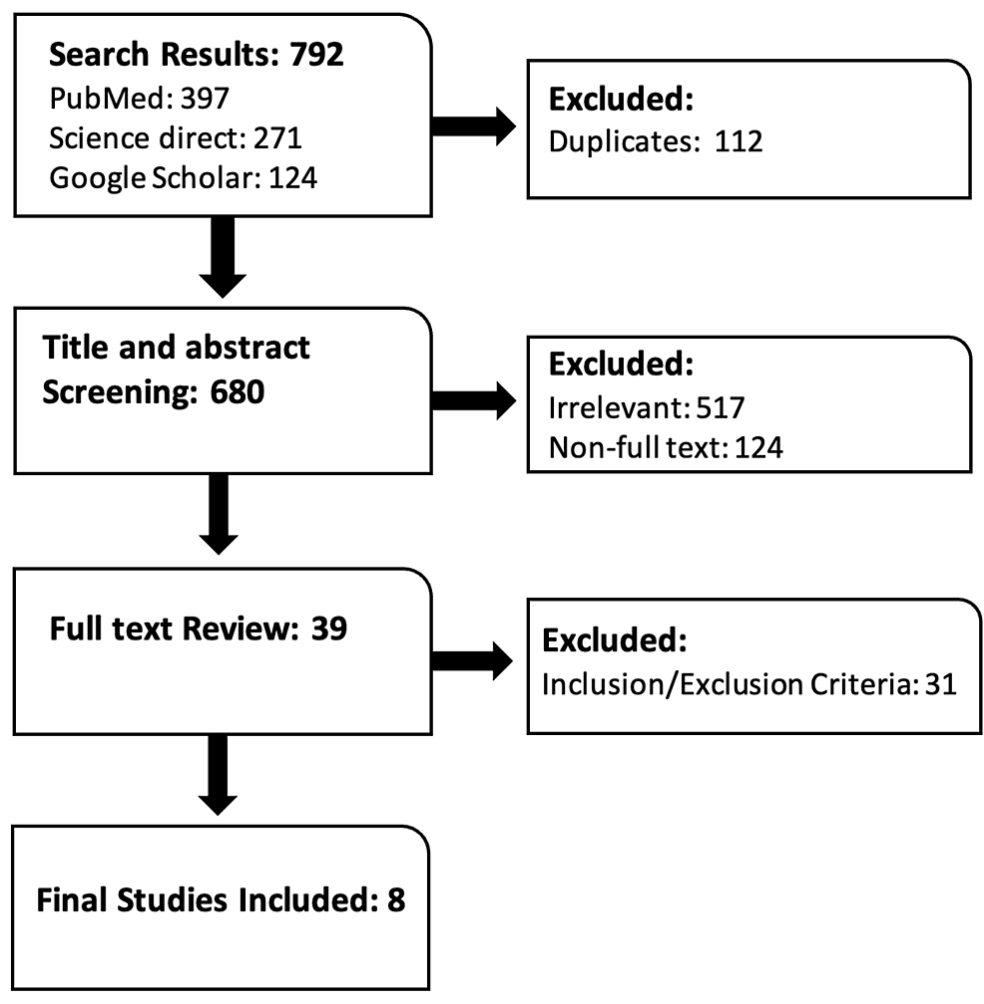
Flow chart showing search results

Figure 2 illustrates the search results of all the databases done from November 26, 2021, to December 5, 2021, as well as the screening procedure for articles, along with the work up to the final eight studies included in this review.

Table 1 below summarizes the key findings of the included studies.

**Table 1:** Summary of the included studies Reg3g: regenerating islet-derived gene γ; cMSCs: canine mesenchymal stromal cells; mRNA: messenger ribonucleic acid; MHC: major histocompatibility complex; NOD: non-obese diabetic; Pdx1: pancreatic and duodenal homeobox 1; Ngn3: neurogenin 3; MafA: V-maf musculoaponeurotic fibrosarcoma oncogene homolog A; IGF1: insulin like growth factor 1; AAV: adeno associated virus; Ngn3-Btc: neurogenin 3- betacellulin; anti-TCRβ mAb: anti-T cell receptor β chain monoclonal antibody; Treg: T regulatory cells

Author	Year	Key Findings
Xia et al. [7]	2015	Lentiviral vector-encoding Reg3g contributes to β cell regeneration and prevents β cells from autoimmune destruction by strengthening regulatory T-cells and generating highly resistant dendritic cells.
Gautham et al. [8]	2016	Targeted lentiviral transduction of the insulin gene into primary cMSCs makes these cells capable of secreting insulin in adequate quantities in vitro, indicating that they could be used in insulin gene therapy.
Fishman et al. [9]	2017	In vivo, mRNA-transfected T-cells expressing chimeric MHC complexes can selectively immunotarget pathogenic T-cells, thus preventing or minimizing the incidence of autoimmune diabetes in NOD mice.
Matsuoka et al. [10]	2017	Pdx1's ability to promote β-cell development from Ngn3-positive endocrine precursors was discovered to be potentiated by MafA, as well as Pdx1's ability to develop β-cells from α-cells.
Mallol et al. [11]	2017	Transgenic NOD mice overexpressing IGF1 specifically in β-cells (NOD-IGF1) and IGF1-encoding AAV of serotype 8 (AAV8-IGF1-dmiRT) treated NOD mice exhibited significantly reduced islet infiltration, preserved β-cell mass, and normalized insulin levels than controls.
Xie et al. [12]	2017	In overtly diabetic mice, an integration of Ngn3-Btc gene therapy and anti-TCRβ mAb treatment resulted in the development of periportal insulin-producing cells in the liver.
Yeh et al. [13]	2017	Transduced lentivirus Treg avatars are more likely to clear islet infiltration/inflammation and contribute to sustained engraftment in the long run.
Xiao et al. [14]	2018	The pancreatic duct was infused with adeno-associated virus containing Pdx1 and MafA expression cassettes in both β cell-toxin-induced diabetic mice and autoimmune NOD animals which then converted α cells into functional β cells and restored blood glucose that persisted for four months prior to reestablishment of autoimmune T1D.

## Review

This section of the review discusses the key findings and highlights of the included studies, the limitations, as well as the feasibility of implementing the techniques used in each study for further enhancing gene therapy in the coming years. The objective of all studies was to revitalize the function of pancreatic β cells that have been adversely affected by the immune system and prevent further destruction of insulin-producing cells [7-14]. 

The Xia et al. study used the lentiviral vector encoding regenerating islet-derived gene γ (Reg3g) in non-obese diabetic (NOD) mice, which showed increased liver-derived alpha antitrypsin-1 (AAT-1) production through Janus kinase (JAK)2/signal transducer and activator of transcription (STAT) 3 signaling pathway. The AAT-1 seemed to possess antiapoptotic and anti-inflammatory properties, thus protecting the β cells in the pancreas from autoimmune destruction while also promoting β cell regeneration leading to suboptimal/optimal insulin levels. They finally proved that in T1D autoimmunity, overexpressed Reg3g might have a role in cell regeneration, decreasing inflammatory reactions, and restoring self-tolerance [7].

Gautham et al. study demonstrated proinsulin gene expression in bone marrow-derived canine mesenchymal stromal cells (cMSCs), permitting these cells to act as proxy beta cells in vitro. Insulin secretion was quantified via the C-peptide levels after primary cMSCs were transduced with a lentiviral vector carrying the furin cleavable proinsulin and green fluorescent protein (GFP) genes. C-peptide levels are deemed a more accurate method of measuring insulin secretion than direct insulin measurements. Once a lentiviral vector has incorporated and expresses a transgene, it is seldom turned off, as evidenced by the sustained level of transgene expression in culture for up to 21 days after transduction, thus establishing adequate insulin secretion in the short and long term [8].

Electroporation is a transfection method used in gene therapy where an electrical pulse is used to generate temporary gaps in cell membranes through which nucleic acids can penetrate the cells [15]. The electroporation of mRNA is a quick, easy, and effective method for delivering mRNA while retaining cell viability [9]. Following the electroporation of mRNA encoding peptide/ β2 microglobulin (β2m)/CD3-ζ, Fishman et al. demonstrated that CD8 T-cells can be reprogrammed to identify diabetogenic T-cells and that this might target autoreactive CD4 and CD8 T-cells (CTLs) in vivo to reduce insulitis and alleviate autoimmunity in the NOD mouse. The T-cell receptor (TCR) complex relies on the CD3-ζ chain for signaling. T-cells can target peptide-specific CD8 T-cells when they are genetically steered by MHC-I heavy (α) chains fused with CD3- ζ and supplemented with a peptide of choice, which is first achieved through the activation of MHC-Iα/CD3-ζ fusion proteins. When insulin B chain, amino acids 15-23 (InsB15-23)/β2m/CD3-ζ or islet-specific glucose-6-phosphatase catalytic subunit-related protein, amino acids 206-214 (IGRP206-214)/β2m/CD3-ζ mRNA-transfected cells were transferred to young NOD female mice, diabetes protection was only seen when the cells targeted InsB15-23-reactive T-cells. The study also demonstrated that introducing the cells at an early age increases targeting insulin-reactive T-cells [9].

The pancreatic acinar cells can be reprogrammed into β cell-like cells by ectopic expression of a combination of three important pancreatic β cell transcription factors, namely pancreatic and duodenal homeobox 1 (Pdx1), neurogenin 3 (Ngn3), and v-maf musculoaponeurotic fibrosarcoma oncogene homolog A (MafA) [10]. Pdx1, a transcription factor required for β cell maturation, proliferation, and function in the pancreas, and MafA is a transcription factor that regulates insulin expression and β cell metabolism by binding to the insulin promoter [14].

The Matsuoka et al. study looked into the idea of reprogramming other pancreatic native cells to insulin-producing β cells. They concluded that Islet β-cell-enriched MafA could enhance the ability of Pdx1 to convert Ngn3-positive cells to insulin-positive cells while also allowing Pdx1 to transform α cells into β cells [10]. Parallel research by the Xiao et al. team proved that: 1) in vivo, Pdx1, and MafA expression reprograms mouse α cells into β cells, 2) normalization of β-cell toxin-induced diabetic mice was achieved using reprogrammed β cells, 3) delayed onset diabetes was seen in autoimmune NOD mice due to reprogrammed β cells, and finally, 4) in vitro, Pdx1, and MafA expression reprogrammed human α cells into β cells. As a result, it could be a new therapeutic method to boost endogenous insulin synthesis, perhaps in combination with immunosuppression. This study mainly focused on using the α cells due to their similarity to β cells (an endocrine cell), the rampant availability, and the easy accessibility because of their proximity to the islet. Furthermore, the partial reduction in α cell mass due to their conversion to β cells may be beneficial in achieving better blood glucose control. Figure 1 depicts the process of reprogramming α cells into β cells through gene therapy [14]. 

**Figure 3:**
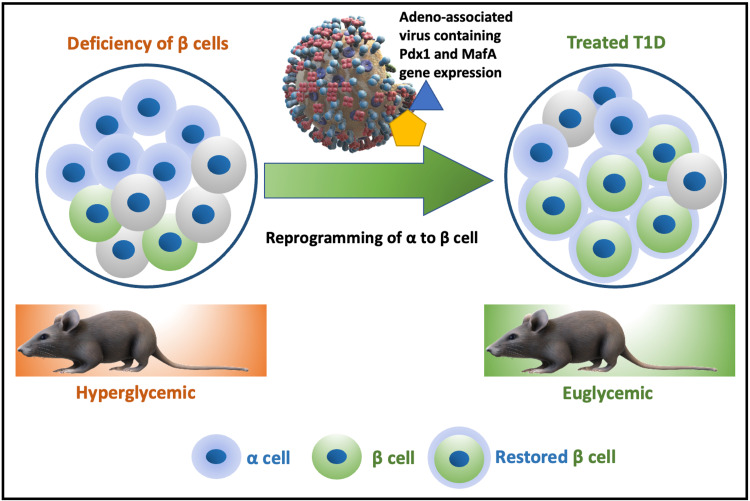
Reprogramming α cells into β cells through gene therapy

Mallol et al. discovered that local insulin-like growth factor-1 (IGF-1) production prevents NOD mice from spontaneous immune-mediated β cell loss and impedes hyperglycemic episodes, first in a transgenic animal model and later using adeno associated virus (AAV) mediated pancreatic gene transfer. Finally proving, gene therapy that specifically overexpresses IGF-1 in the pancreas controls disease progression in T1D [11].

The Xie et al. study focused on anti-T cell receptor β chain monoclonal antibody (anti-TCRβ mAb) plus neurogenin 3- betacellulin (Ngn3-Btc) gene transfer for the reversal of T1D while impeding islet destruction through selective elimination of diabetogenic T-cells while preserving the other components of the immune system. A five-day course of 50 μg of anti-TCR β mAb reversed >80% of new-onset T1D in NOD mice, and close to 60% reversal was seen in anti-TCRβ mAb resistant overtly diabetic NOD mice after treating them with a combination of anti-TCRβ mAb and Ngn3-Btc gene transfer. Furthermore, in animal models, an anti-TCRβ mAb clone was shown to inhibit experimental autoimmune encephalomyelitis and collagen-induced arthritis [12].

Yeh et al. investigated the use of lentiviral gene transfer of TCRs that detect type 1 diabetes-related autoantigens. The primary goal of this research was to inhibit β-cell destruction by inducing antigen-specific tolerance in a tissue-specific manner. Human T regulatory cells (Tregs) were cultured that produced both a high-affinity glutamic acid decarboxylase 555-567 (GAD555-567) reactive TCR (clone R164) and a lower affinity clone 4.13 specific for the same peptide [15]. Tregs effectively reduce antigen-specific and bystander responder T-cell (Tresp) proliferation in vitro. The high-affinity R164 Tregs suppressed autoantigen more effectively than the lower-affinity 4.13 Tregs. As a result, avatar Tregs may reduce β-cell autoimmunity and highlight new prospects for cellular engineering specificities and phenotypes to regulate Treg activity in T1D adoptive cell therapy [13].

Limitations

This review mainly emphasizes animal and in vitro studies focusing on regenerating insulin-producing cells and does not address other hormones involved in glucose metabolism, such as incretins. More studies are required to comprehend the relationship between insulin secretion through gene therapy with other endocrine parameters involved in the pathogenesis of T1D. Ex vivo insulin gene therapy has been limited by the lack of an optimal cell type that can be easily acquired, changed to make insulin, and reimplanted. Gene therapy by itself requires ongoing research to address the risks such as cancer, toxicity, and inflammation.

## Conclusions

In summary, gene therapy has been around for more than two decades, and various researches are ongoing in this field daily. The studies included in this review applied gene therapy for T1D in multiple modalities and demonstrated preservation of pancreatic β-cells, thus optimizing insulin secretion levels, while few studies also focused on sustaining this reference range insulin secretion for months, contributing to look about the long-term benefits. Targeted viral vector (lentivirus or adenovirus) transduction or gene expression in the interest of regenerating β-cells and mRNA transfected T-cells targeting insulin-reactive CD8 T cells aid in preventing T1D. Another approach is through gene transfer with a combination of anti-TCRβ mAb with Ngn3-Btc to produce insulin-producing cells in the liver. These are some of the evolving methods for promising gene therapy seen in the recent decade. The majority of the recent studies are done on animals or as preclinical trials; however, with the growing understanding of gene therapy, may one day lead to a cure for autoimmune diseases like T1D, and further research needs to ensure the large-scale benefits, especially in vivo studies in a human population of interest.
